# Detection of disease recurrence in real-world patients with completely resected stage III cutaneous melanoma

**DOI:** 10.2340/1651-226X.2026.45609

**Published:** 2026-06-08

**Authors:** Tiina Peuranheimo, Anni Hellman, Siru Mäkelä, Kalle E. Mattila, Micaela Hernberg

**Affiliations:** Comprehensive Cancer Center, Helsinki University Hospital, Helsinki, Finland

**Keywords:** melanoma, cutaneous malignant, tomography, X-ray computed, whole body imaging, neoplasm metastasis, neoplasm recurrence, follow-up studies

## Abstract

**Background and purpose:**

Optimal postoperative follow-up for high-risk cutaneous melanomas (CM) is unclear. We aimed to evaluate disease recurrences in patients with stage III CM before modern adjuvant therapies.

**Patient/material and methods:**

Three hundred and fifty patients with a median follow-up of 8.9 years at Helsinki University Hospital Comprehensive Cancer Center after complete resection of stage IIIA–D CM between 2008 and 2017 were identified. Information on baseline characteristics, recurrence patterns and survival were collected from electronic medical records.

**Results:**

One hundred and ninety patients (54.3%) developed disease recurrence with 258 recurrence events documented. Local recurrence occurred in 14.5, 27.0, 32.3 and 44.4% and distant metastases in 21.7, 40.5, 52.4 and 83.3% of patients with stage IIIA, IIIB, IIID and IIID, respectively. Median recurrence-free survival was not reached and 7.4 years in stage IIIA and IIIB compared to 2.9 and 0.9 years in IIIC and IIID. Routine computed tomography (CT) imaging revealed 51% of all recurrences and 64% of distant metastases. 13.2% of distant recurrences involved skin and soft tissues, 31.4 lungs, 34.0 other visceral organs and 21.5% brain. First recurrences occurred mostly during the first 2 years of follow-up (59.5–100% in stage IIIB–D) except for stage IIIA (45.0%). Most of recurrences detected by patients (58.0%), physical exams or ultrasound (76.1%) and routine CT (61.2%) occurred during the first 2 years.

**Interpretation:**

High rate of recurrences and distant metastases supports routine physical exams and CT particularly during the first years of postoperative follow-up, especially in stage IIIB–D CM.

## Introduction

The incidence of cutaneous melanoma (CM) is increasing in Finland and in other developed countries with predominantly fair-skinned inhabitants [1, 2]. It is estimated that melanomas will represent 5.1% of all new cancer cases in the US in 2025 [3]. However, mortality rates have decreased probably due to earlier detection of primary melanomas and effective systemic treatment options in patients with metastatic and completely resected high risk melanomas in all age groups [4–10].

After excision of the primary tumour, sentinel lymph node biopsy (SLNB), and complete lymph node dissection (CLND), the latest classification of American Joint Committee of Cancer (AJCC 8th edition) stratifies patients according to the risk of death from melanoma [11]. Approximately 10% of primary CMs are diagnosed with regional lymph node metastasis (stage III) [11, 12]. Currently, the 5-year survival rate of all patients with melanoma is close to 95% but decreases to 75% in patients with stage III [3]. However, 10-year melanoma-specific survival rates within stage III CM range from 88% in patients with stage IIIA to 24% in stage IIID depending on thickness and ulceration of the primary tumour, the presence of in-transit metastases and the extent of lymph node involvement [11, 13]. Currently, adjuvant treatment with Programmed death ligand 1(PD-L1) inhibitors or v-raf murine sarcoma viral oncogene homolog B1(BRAF) and Mitogen-activated protein kinase(MEK) inhibitors is used to decrease the risk of recurrence after complete resection of stage III CM [5]. Recent studies have shown that perioperative immunotherapy is more effective than adjuvant immunotherapy for patients with clinically detected regional lymph node metastases [14–16].

Postoperative follow-up with physical exams and routine cross-sectional imaging (CSI) with thoracic and abdominal computed tomography (CT) or positron emission tomography (PET-CT) ± brain CT or magnetic resonance imaging (MRI) is recommended for patients from stage IIB to IV by the European Society For Medical Oncology (ESMO) guidelines [5]. However, there are variable practices in different countries and different hospitals, particularly considering the use of imaging [17]. There are both retrospective and prospective studies supporting the use of routine CSI during postoperative follow-up for high-risk patients to detect asymptomatic disease recurrences early [18–21] although conflicting results are also presented [22]. Surgery is an option for locoregional recurrences, and some patients might be candidates for resection of solitary distant metastases potentially leading to survival benefit even in the era of modern systemic therapies [23]. In unresectable and metastatic CM, it is observed that lower tumour burden yields better responses and longer survival with immunotherapy and targeted therapies [24–27].

In this study, we aimed to evaluate the risk, timing, site and detection method of recurrence among patients with stage IIIA–IIID CM undergoing postoperative follow-up at Helsinki University Hospital Comprehensive Cancer Center in a cohort before the use of PD-1 inhibitors, and targeted therapy in adjuvant and neoadjuvant settings was standard of care.

## Patients/material and methods

### Study design and patient selection

This study was a retrospective register study. All patients (*n* = 777) admitted to follow-up at Helsinki University Hospital Comprehensive Cancer Center after surgery for stage IIB–IV CM between 2008 and 2017 were identified from electronic medical records. Baseline patient characteristics (age and sex), histological tumour characteristics (e.g. Breslow thickness, ulceration and BRAF mutational status), surgical procedures, results of perioperative CSI and adjuvant therapies were collected, and patients were restaged according to the AJCC 8th edition by study investigators (TP, AH).

**Figure 1:**
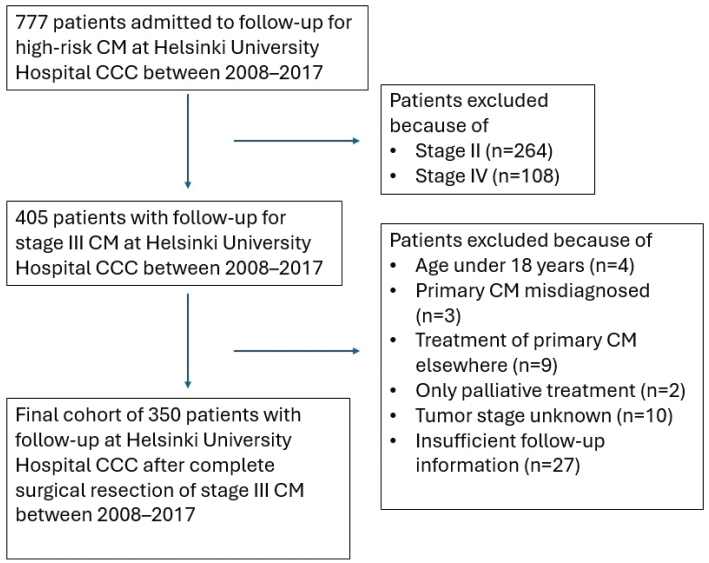
Patient selection flowchart. CM: cutaneous melanoma; CCC: Comprehensive Cancer Center.

Patient selection for the final study cohort is described in Figure 1. Four hundred and five patients had positive SLNB or had undergone CLND for stage III CM. Patients classified as having stage III CM had to be disease-free according to thoracic and abdominal CT ± brain CT or MRI scans before or after surgery. Patients with suspicious findings on perioperative CT scans that were later confirmed metastatic in subsequent imaging were classified as having stage IV CM and were excluded from the final study cohort. After excluding also patients under 18 years of age, patients with primary CM misdiagnosed, patients with the treatment of primary CM outside Helsinki University Hospital district, patients with palliative treatment only, patients with unknown tumour stage and patients with insufficient follow-up information, 350 patients with completely resected stage III CM were included into the final study cohort (Figure 1).

### Postoperative follow-up

For patients included in the final study cohort, sites of disease recurrence (local recurrences determined as local, in-transit and regional lymph node recurrences or distant recurrences), detection methods, dates of recurrence, survival status and causes of death were collected from electronic medical records by study investigators (TP and AH) until 29 February 2024. Recurrences were registered as ‘detected by patient’ if patient first noticed new symptoms, skin lesions or palpable lymph nodes. Recurrences found by routine physical exams, ultrasounds (US) or CT imaging performed according to institutional follow-up protocols were also determined. Routine US was mainly performed for patients taking part to the Multicenter Selective Lymphandenectomy Trial 2 (MSLT-2)- study (*n* = 72). Although our follow-up program for patients with high-risk CM evolved slightly during the study period, annual thoracic and abdominal CT scans were performed throughout study period, whereas routine brain imaging was not included for all patients. Details of the postoperative follow-up protocols for patients with high-risk CM during the study period are described in Supplementary Figure 1.

### Statistical methods

Categorical variables were presented as numbers and percentages and continuous variables with median and range. Statistical differences were assessed using Pearson’s Chi-squared test (categorical variables) and Kruskal–Wallis (continuous variables), and corresponding *p*-values were reported. The median follow-up time with 95% confidence interval (CI) was calculated using the reverse Kaplan–Meier method. The Kaplan–Meier method was used to calculate median recurrence-free survival (RFS), and distant metastasis-free survival (DMFS) estimates with 95% CI, and the Log Rank test was used to analyse statistical significance between subgroups (stage IIIA–D). RFS was calculated from the diagnosis of primary CM to local or distant recurrence (event), death from melanoma (event), death from other causes (censored) or the last visit (censored). DMFS was calculated from the diagnosis of primary CM to distant metastases (event), death from melanoma (event), death from other causes (censored), or the last visit (censored). Overall survival (OS) was calculated from the primary CM diagnosis to death from melanoma (event), death from any cause (event) or the last visit (censored). Melanoma-specific survival (MSS) was calculated from date of diagnosis of primary CM to death from melanoma (event), death from any cause (censored) or the last visit (censored). Cumulative percentages of patients being event-free at the end of each year of follow-up were calculated using the Life-table method. Statistical analyses were performed with Statistical Package for the Social Science (SPSS) 29 v100 and R (version 4.5.1.).

## Results

### Baseline characteristics

Baseline characteristics of all 350 patients with completely resected stage III CM are described in Table 1. The median age was 61 years (range 18–86). One hundred and ninety-five patients (55.7%) were males and 155 (44.3%) females. According to AJCC 8th classification, 69 patients (19.7%) had stage IIIA, 74 (21.1%) stage IIIB, 189 (54.0%) stage IIIC and 18 (5.1%) stage IIID CM. In addition to wide excision of the primary tumour, 287 patients (82.0%) had undergone CLND and 63 patients (18.0%) SLNB only. One hundred patients (28.7%) received adjuvant therapy, which was most commonly radiotherapy (56 patients) or interferon alpha (37 patients) (Table 1).

**Table 1:** Patient characteristics.

Characteristics	All patients 350 (100%)	Stage IIIA 69 (19.7%)	Stage IIIB 74 (21.1%)	Stage IIIC 189 (54.0%)	Stage IIID 18 (5.1%)
**Median age, years (range)**	61.7 (18.6–86.7)	55.8 (18.6–83.2)	58.2 (23.1–85.1)	63.0 (20.1–86.7)	71.8 (56.3–85.4)
**Sex**					
Female	155 (44.3%)	32 (46.4%)	38 (51.4%)	74 (39.2%)	11 (61.1%)
Male	195 (55.7%)	37 (53.6%)	36 (48.6%)	115 (60.8%)	7 (38.9%)
**Median Breslow (range)**	2.9 (0.3–37.0) mm	1.2 (0.6 –2.0) mm	2.3 (0.3–4.0) mm	4.1 (1.2–37.0) mm	5.8 (4.2–17.0) mm
**Operation**					
SLNB only	63 (18.0%)	14 (20.3%)	12 (16.2%)	37 (19.6%)	0 (0%)
CLND	287 (82.0%)	55 (79.7%)	62 (83.8%)	152 (80.4%)	18 (100%)
**BRAFV600**					
Positive	127 (36.3%)	16 (23.2%)	30 (40.5%)	73 (38.6%)	8 (44.4%)
Negative	104 (29.7%)	13 (18.8%)	26 (35.1%)	57 (30.2%)	8 (44.4%)
Unknown	119 (34.0%)	40 (58.0%)	18 (24.3%)	59 (31.2%)	2 (11.1%)
**Adjuvant therapy**					
Any adjuvant therapy	100 (28.7%)	5 (7.2%)	18 (24.3%)	67(35.6%)	10 (55.6%)
Radiation therapy	49 (14.0%)	0 (0.0%)	8 (10.8%)	31 (16.5%)	10 (55.6%)
Medical therapy^a^	44 (12.7%)	5 (7.2%)	9 (12.2%)	30 (16.0%)	0 (0.0%)
Radiation and medical^b^	7 (2.0%)	0 (0.0%)	1 (1.4%)	6 (3.2%)	0 (0.0%)

SLNB: sentinel lymph node biopsy; CLND: complete lymph node dissection.

a Thirty-seven patients had interferon-alpha, four had ipilimumab and three patients had pembrolizumab/placebo;

b Patients who had both radiation therapy and interferon-alpha as an adjuvant treatment.

### Recurrence rates

**Figure 2:**
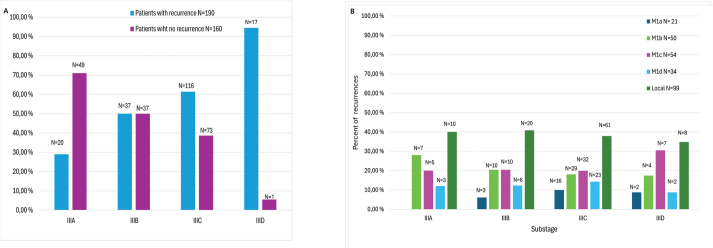
Recurrence rates for stages IIIA–IIID (panel A) and distribution of local and distant recurrences as percentage for each substage (panel B). Absolute number of recurrences are presented above columns. M1a, distant metastasis to skin, soft tissue including muscles, and/or nonregional lymph node; M1b, distant metastasis to lung; M1c, distant metastasis to non-central nervous system(CNS) visceral sites; M1d, distant metastasis to CNS.

After the median postoperative follow-up of 8.9 years (95% CI 7.6–10.1), 190 patients (54.3%) had developed disease recurrence. Recurrence rates increased from stage IIIA to IIID. Twenty (29.0%) patients with stage IIIA, 37 (50.0%) patients with stage IIIB, 116 (61.4%) patients with stage IIIC, and 17 (94.4%) patients with stage IIID CM developed disease recurrence, *p* < 0.001 (Figure 2A).

### Sites of recurrence

During postoperative follow-up, local recurrence was observed in 99 patients (28.3%) and distant recurrence in 159 patients (45.4%) (Figure 2B). Eighteen (5.1%) patients had synchronous local and distant recurrences (defined as the detection of local and distant recurrence at the same time point), whereas 50 (14.3%) patients had metachronous recurrences (sequential detection at different time points) (Supplementary Table 1).

The rate of local recurrences increased significantly with advancing stage and occurred in ten (14.5%) patients with stage IIIA, 20 (27.0%) patients with stage IIIB, 61 (32.3%) patients with stage IIIC and eight (44.4%) patients with stage IIID, *p* = 0.016 (Figure 2B). Similarly, distant metastases were detected in 15 (21.7%) patients with stage IIIA, 30 (40.5%) patients with stage IIIB, 99 (52.4%) patients with stage IIIC and 15 patients (83.3%) with stage IIID, *p* < 0.001 (Figure 2B). The rate of local recurrences was higher in patients with only SNLB (36.5%) compared to patients with CLND (26.5%) *p* = 0.110 (Supplementary Table 1).

Overall, 81 patients (23.1%) presented with local recurrence as the first manifestation of melanoma relapse, 91 (26.0%) with distant recurrence and 18 (5.1%) with both local and distant recurrence (Supplementary Table 1). The proportion of distant recurrence as the initial site of recurrence increased progressively from stage IIIA to IIID (14.5–50.0%), *p* < 0.001 (Supplementary Table 1).

### RFS and DMFS

**Figure 3:**
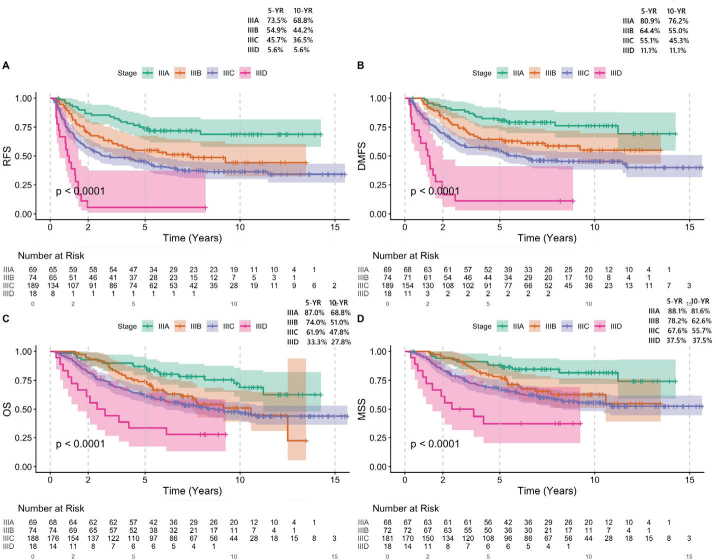
Recurrence-free survival (panel A) and distant metastasis-free survival (panel B), overall survival (panel C) and melanoma-specific survival (panel D). RFS: recurrence free survival; DMFS: distant metastasis free survival; OS: overall survival; MSS: melanoma-specific survival.

The median RFS was 5.2 years (3.3–7.1) for all patients. Median RFS was not reached in patients with stage IIIA, 7.4 years (2.2–12.6) in stage IIIB, 2.9 years (1.0–4.8) in stage IIIC and 0.9 years (0.6–1.3) in stage IIID, respectively, *p* = < 0.001 (Figure 3A). Median DMFS was not reached in patients with stage IIIA and stage IIIB, 6.0 years (2.1–10.0) in stage IIIC and 1.2 years (0.8–1.6) in stage IIID, *p* = < 0.001 (Figure 3B).

### OS and MSS

The median OS was 10.5 years (8.0–12.9) for all patients. The median OS was not reached in stage IIIA, 10.6 years (6.6–14.6) in stage IIIB, 8.7 years (6.0–11.4) in stage IIIC and 2.5 years (0.8–4.1) in stage IIID, *p* = < 0.001 (Figure 3C). The median MSS was not reached in stages IIIA–C and was 2.5 years (0.0–5.5) in stage IIID, *p* = < 0.001 (Figure 3D). The 5-year and 10-year MSS rates were 88.1 and 81.6% for stage IIIA, 78.2 and 62.6% for stage IIIB, 67.6 and 55.7% for stage IIIC and 37.5 and 37.5% for stage IIID (Figure 3D).

### Timing of first recurrence

**Figure 4:**
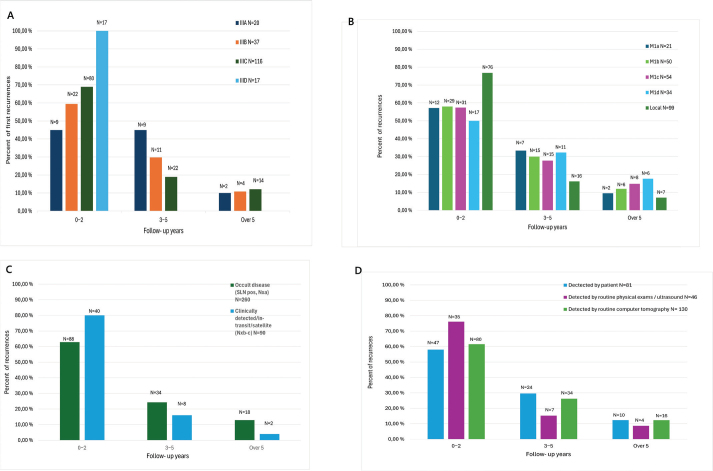
Distribution of first recurrences as percentage for stages IIIA–IIID (panel A), distribution of local and distant recurrences as percentage for stages IIIA–IIID (panel B), distribution of recurrences for patients with occult disease (sentinel lymph node[SLN] pos, Nxa) or clinically detected/in-transit/satellite disease (Nxb–c) (panel C) and detection methods (panel D) for follow-up years 0–2, 3–5 and over 5 years. Absolute number of recurrences are presented above each column. M1a, distant metastasis to skin, soft tissue including muscles, and/or nonregional lymph node; M1b, distant metastasis to lung; M1c, distant metastasis to non-CNS visceral sites; M1d, distant metastasis to CNS.

67.4% (*n* = 128) first recurrences were detected during the first 2 years of follow-up, whereas 20 10.5% (*n* = 20) first recurrences were detected after 5-year follow-up period in our institute (Figure 4A). The proportion of first recurrences detected within the first 2 years increased with advancing stage, and was 45.0% in patients with stage IIIA, 59.5% in stage IIIB, 69.0% in stage IIIC and 100.0% in stage IIID (Figure 4A).

### Timing of local and distant recurrences

76.8% (*n* = 76) of all local recurrences were detected during the first 2 years of follow-up, 16 16.2% (*n* = 16) between years 3–5 and 7.0% (*n* = 7) after 5-year follow-up period (Figure 4B). Twenty-one (13.2%) of 159 distant recurrences involved skin or soft tissue (M1a), 50 (31.4%) lungs (M1b), 54 (34.0%) other visceral organs (M1c) and 34 (21.5%) brain (M1d). Rates of distant recurrences detected in the first 2 years of follow-up were 57.1% (*n* = 12) in M1a, (58.0% (*n* = 29) in M1b, 31 57.4% (*n* = 31) in M1c, and 50.0% (*N* = 17) in M1d (Figure 4B).

### Timing of recurrences for Nxa and Nxb-b

Two hundred and sixty (74.3%) patients had clinically occult disease, Nxa, and 90 (25.7%) patients had clinically detected lymph node metastases, in-transit metastases or satellites at the time of initial melanoma diagnosis, Nxb–c. 62.9% (*n* = 88) of recurrences for patients with Nxa were detected during first 2 years of follow-up and 80% (*n* = 40) for patients with Nxb–c (Figure 4C).

### Detection methods

Thirty six (36.4%) of local recurrences were detected by patient, 33 (33.3%) by routine physical exams/US and 30 (30.3%) by routine CT. Respectively, 45 (28.3%) of distant recurrences were detected by patient, 13 (8.2%) by routine examination/US and 101 (63.5%) by routine CT. Distribution of detection methods for each stage IIIA–IIID and patients with SLNB and CLND are presented in Supplementary Table 2. Most of the recurrences detected by patient (58.0%), by routine physical exams or US (76.1%) and by routine CT (61.2%) occurred during the first 2 years of follow-up (Figure 4D).

## Discussion and conclusion

In this retrospective cohort of 350 patients with stage III CM treated before modern adjuvant therapies at Helsinki, Finland, 190 patients (54%) developed disease recurrence during the median postoperative follow-up of 8.9 years with a total of 258 recurrence events documented. Not surprisingly, rates of both local (15–44%) and distant recurrences (22–83%) increased significantly from stage IIIA to IIID. Median time to recurrence was longer in patients with stage IIIA and IIIB (not reached [NR] and 7.4 years) compared to stage IIIC and IIID (2.9 and 0.9 years). Most of the first recurrences (67%) were detected within the first 2 years of follow-up. This pattern was also consistent for all recurrences and across different detection methods: 58% of patient-detected recurrences, 76% recurrences detected by routine physical exams or US and 61% of those detected by routine CT imaging were detected during the first 2 years of follow-up.

Although European guidelines recommend routine physical exams and CSI after surgery of high-risk CM [5], there are different follow-up strategies even in the Nordic countries with similar publicly funded healthcare systems. Danish and Norwegian national guidelines include routine whole-body FDG-PET-CT scans in patients with stage IIB to IIID melanomas [12, 28], whereas there has been no repeated CT or FDG-PET-CT scanning in the routine follow-up for high-risk melanomas in Sweden [22]. In Finland, follow-up protocols in different hospital have followed the ESMO guidelines [5] with annual routine thoracic and abdominal CT scans until 5-years from surgery with baseline brain CT/MRI to detect asymptomatic metastases in high-risk patients.

In this cohort of Finnish patients with stage III CM, stage-specific recurrence rates (ranging from 29% in stage IIIA to 94% in IIID) were higher than observed in stage III CM in Denmark (25–71%) [12]. Among patients with stage IIIB–IIID, most of the recurrences were detected within the first 2 years of postoperative follow-up, both in the Danish cohort (57–98%) and in the present study cohort (60–100%). In contrast, only 42% of patients with stage IIIA in the Danish cohort and 45% of patients with stage IIIA in our cohort experienced recurrence within the first 2 years [12]. Since these patients are excluded from modern adjuvant therapies, longer follow-up for this subgroup of patients could be considered as well as for patients who had received modern adjuvant therapies potentially postponing recurrences. Notably, 11% of recurrences in our cohort were detected after 5 years of follow-up, which, together with the 3 year longer median follow-up, may partly explain higher rates of recurrences in our study. Both Finnish and Danish real-world cohorts were treated before the use of PD-1 inhibitors or targeted therapy in the adjuvant/neoadjuvant setting probably explaining slightly higher recurrence rates (54% in the present study) than observed in clinical trials with adjuvant nivolumab (5-year RFS 50%) [29], pembrolizumab (7-year RFS 50%) [30] and Dabrafenib plus Trametinib (5-year RFS 52%) [31] in patients with stage III CM. The 5-year MSS for stages IIIA–IIID was rather similar in our study (88–38%) compared to International Melanoma Database and Discovery Platform (93–32%) and compared to other Nordic cohorts [12, 32].

In our study, the rate of distant recurrence was 45%, and most distant metastases were detected by routine follow-up CT imaging (64%) before being symptomatic and detected by patients or physical exams. These results are comparable to earlier studies with 57–75% rates of distant recurrences detected by routine imaging [15–18, 28]. Most of the distant recurrences in all M-stages were detected during first years of follow-up. 61% of recurrences detected by routine CT were detected within the first 2 years of follow-up.

The limitations of this study are attributed to its retrospective design and single-centre patient cohort treated before modern adjuvant therapies. However, all patients with high-risk CM at our region were admitted to postoperative follow-up at Helsinki University Hospital Comprehensive Cancer Center decreasing the risk of selection bias. To our knowledge, there are only few reports describing stage-specific sites of recurrences and detection methods in detail. During the study period, institutional follow-up protocols evolved slightly, but annual CT scans and outpatient visits for 5 years were performed throughout the study period. As patients were treated before the use of PD-1 inhibitors and targeted therapy in the adjuvant setting, our results are not directly applicable to patients receiving modern neoadjuvant and adjuvant therapies, potentially postponing recurrences. In this study, the 2-year RFS for stages IIIB–IIID was approximately 57% which was lower than observed in adjuvant trials with PD-1 inhibitors and targeted therapies with 2-year RFS rates ranging from 65 to 70% [29–31]. Moreover, most patients in this study had undergone CLND and received postoperative radiotherapy reflecting treatment practices before MSLT-2 study. It was observed that the rate of local recurrences was higher among SLNB only patients compared to CLND patients. Despite the high rate of CLNDs, the rate of local recurrences (28%) was higher in this study compared to 16.3–17.3% seen in the observational arms of the Dermatologic Cooperative Oncology Group (DeCOG)-SLT and the MSLT-2 studies [33, 34]. However, most recurrences were distant (61.6%) as observed in earlier studies [12, 19–21] supporting the generalizability of our findings in patients with stage III CM.

As 51% of all recurrences and 64% of distant metastases were detected with routine CT before signs and symptoms, our results strengthen the rationale for repeated imaging during postoperative follow-up of patients with high-risk CM. Whether it translates into survival benefit remains still debatable [22]. Notably, in the TRIM study, patients receiving adjuvant treatment in both groups (27 and 28%) underwent whole-body imaging at 6 months from treatment initiation, and all patients were monitored with lymph node US surveillance. This should be taken into account when interpreting these results. Early detection of metastatic disease with routine imaging may spare patients from toxicities of the treatment and decrease costs, especially as it is known that patients treated in routine clinical practices may differ from those treated in clinical trials [35].

## Conclusions

Although it is uncertain whether early detection of asymptomatic recurrences translates into survival benefit, more than half (54%) of patients with completely resected stage III CM developed disease recurrence during a median follow-up of 8.9 years, most commonly during the first 2 years of follow-up. Routine follow-up CT imaging detected 51% of all recurrences and 64% of distant recurrences, suggesting a role of routine CSI at least during the first years of postoperative follow-up for stage III CM.

## Supplementary Material



## Data Availability

Clinical data included in this study are not publicly available.
