# Supplementary material for: Detection of disease recurrence in real-world patients with completely resected stage III cutaneous melanoma
Source: Acta Oncol. 2026 Jun 8;65:45609. doi: 10.2340/1651-226X.2026.45609 (PMC13248745; doi:10.2340/1651-226X.2026.45609)
Supplement: Supplementary file 1 [file AO-65-45609-s1.pdf]

Supplementary material has been published as submitted. It has not been copyedited, or typeset by Acta Oncologica

Supplementary figure 1. Follow-up program of resected cutaneous melanoma patients in Helsinki University Hospital Comprehensive Cancer Center in 2008 and 2024.

2008 ->

Stage IIBC, stage III patients*:

First follow up visit: Body CT, lab***., and clinical examination

Year 1 –2: clinical examination** every three months.
lab***. and body CT at 6,12,18, 24 months

Year 3 – 5: clinical examination every 6 months
lab***. and body CT at 3, 4 and 5 years

2024->

Stage IIBC, III, IV resected, (no evidence of disease), no adjuvant treatment:

First follow- up visit: lab****; body CT and clinical examination

Year 1: lab; body CT and clinical examination at 6 and 12 months

Year 2: clinical examination yearly
lab****, and body CT at 18 and 24 months

Year 3–5: clinical examination yearly
lab****, and body CT at 3,4 and 5 years

Stage IIC, III BCD, IV resected (no evidence of disease), adjuvant treatment:

First follow- up visit: body and brain CT, clinical examination, lab.****

Year 1: clinical examination every four months
lab****; body and brain CT at 4, 8 and 12 months

Year 2: clinical examination yearly
lab**** and body CT at 18 and 24 months

3 –5 years: clinical examination yearly
lab****. and body CT at 3,4 and 5 years

CT, computer tomography; lab, laboratory tests,

*For patients having adjuvant interferon alpha treatment, follow-up visits were only in the cancer clinic. After adjuvant treatment and for patients not having adjuvant treatment follow-up visits were in oncology and plastic surgery unit in turns. Patients taking part in the Multicenter Selective Lymphandenectomy Trial 2 (MSLT-2) were followed according to the MSLT2 trial protocol and oncology visits were replaced with phone calls from oncology unit except visits at the beginning of follow-up, 1 year, 3 years and 5 years. Clinical examinations every 4 months took place in the plastic surgery unit.

**For patients having plenty of naevus (z100 >4mm pigmented naevus), melanoma, dysplastic naevus and melanoma, skin examination in the dermatology unit every 6-12 months. For patients having pervious criteria but not family history of melanoma skin examination in the oncology clinic for 5 years.

***basic blood picture, liver enzymes, renal function, C-reactive protein (CRP), lactate dehydrogenase and thyroid-stimulating hormone (TSH) for those who had received irradiation to the neck area.

**** For patients receiving adjuvant treatment, more precise laboratory tests were performed; for those not anymore receiving adjuvant treatment, less precise tests

Supplementary table 1. Stage-specific recurrence rates and recurrences in patients with SLNB only and CLND

	All stage III patients (N= 350)	Stage IIIA (N=69)	Stage IIIB (N=74)	Stage IIIC (N=189)	Stage IIID (N=18)	Patients with SLNB only (N=63)	Patients with CLND (N=287)
Patients with recurrence N (%)	190 (54.3%)	20 (29.0%)	37 (50.0%)	116 (61.4%)	17 (94.4%)	31 (49.2%)	159 (55.4%)
All patients with local recurrence as the 1st presentation N (%)	81 (23.1%)	8 (11.6%)	19 (25.7%)	48 (25.4%)	6 (33.3%)	20 (31.7%)	61 (21.3%)
All patients with distant recurrences as the 1st presentation N (%)	91 (26.0%)	10 (14.5%)	17 (23.0%)	55 (29.1%)	9 (50.0%)	8 (12.7%)	83 (28.9%)
All patients with local and distant recurrences as 1st presentation N (%)	18 (5.1%)	2 (2.9%)	1 (1.4%)	13 (6.9%)	2 (11.1%)	3 (4.8%)	15 (5.2%)
All patients with local recurrences during follow-up N (%)	99 (28.3%)	10 (14.5%)	20 (27.0%)	61 (32.3%)	8 (44.4%)	23 (36.5%)	76 (26.5%)
All patients with distant recurrences during follow-up N (%)	159 (45.4%)	15 (21.7%)	30 (40.5%)	99 (52.4%)	15 (83.3%)	24 (38.1%)	135 (47.0%)

SLNB, sentinel lymph node biopsy; CLND complete lymph node dissection

Supplementary Table 2 Detection methods of disease recurrence during postoperative follow-up

	Detected by patient	Detected by routine physical exams / ultrasound	Detected by routine computer tomography
All recurrences 258 (100%)	81 (31.4%)	46 (17.8%)	131 (50.8%)
Local recurrences 99 (100%)	36 (36.4%)	33 (33.3%)	30 (30.3%)
Distant recurrences 159 (100%)	45 (28.3%)	13 (8.2%)	101 (63.5%)
Recurrences in patients with stage IIIA 25 (100%)	10 (40.0%)	5 (20.0%)	10 (40.0%)
Recurrences in patients with stage IIIB 50 (100%)	15 (30.0%)	11(22.0%)	24 (48.0%)
Recurrences in patients with stage IIIC 160 (100%)	47 (29.4%)	21 (17.5%)	85 (53.1%)
Recurrences in patients with stage IIID 23 (100%)	9 (39.1%)	2 (8.7%)	12 (52.2%)
Recurrences in patients with SLNB only 47 (100%)	19 (40.4%)	13 (27.7%)	15 (31.9%)
Recurrences in patients with CLND 211 (100%)	62 (29.4%)	33 (15.6%)	116 (55.0%)

SLNB, sentinel lymph node biopsy; CLND complete lymph node dissection
